# Effect of waxy barley, Kirarimochi, consumption on bowel movements of late-stage elderly residents at Roken nursing home

**DOI:** 10.1186/s40101-017-0131-0

**Published:** 2017-02-20

**Authors:** Keiko Taniguchi, Kozo Komae, Asuka Takahashi, Toji Yoshioka, Yoshiaki Sone

**Affiliations:** 1 Mimasaka Sakutou Nursing Home, 280 Emi, Mimasaka, Okayama 709-4234 Japan; 2 Present Address: Health promotion section, 390-2 Kitayama, Mimasaka, Okayama 707-0014 Japan; 3 Konan Women’s University, 6-2-23, Morikita, Higashinada-ku, Kobe, Hyougo 658-0001 Japan; 4 NARO Western Region Agricultural Research Center, Crop Breeding and Food Functional Components Research Division, 1-3-1 Senyu-cho, Zentsuji, Kagawa 765-8508 Japan; 5 Graduate School of Human Life Science, Mimasaka University, 50 Kitasono-cho, Tsuyama, Okayama 708-8511 Japan

**Keywords:** Waxy barley, Dietary fiber, Defecation, Laxative administration, Bowel movement, Constipation, Nursing home, Late-stage elderly

## Abstract

**Background:**

It is very important for the late-stage elderly to have the least stressful bowel movements for maintaining a good quality of life. It is generally accepted that consuming adequate dietary fiber is a promising method for the prevention and management of stressful bowel movements such as those during constipation. Therefore, we examined the effect of long-term consumption of waxy barley, which is high in dietary fiber, on the bowel movements of the late-stage elderly living at Roken nursing home (a geriatric health services facility), Japan.

**Methods:**

We compared the defecation and laxative administration frequencies of the subjects before and after waxy barley consumption, for which we served 28 residents a boiled mixture of rice and waxy barley (variety name, Kirarimochi) as the main meals for 5 months, from November to March. In October, all residents were served boiled rice as the main meals.

**Results:**

The residents were categorized into “constipated” subjects and “non-constipated” subjects according to their weekly defecation frequency during October. Among the 14 residents categorized as constipated subjects, monthly number of days with defecation in November, January, and March significantly increased in comparison to monthly number of days with defecation in October. In addition, monthly number of days with laxative administration significantly decreased in December and February in comparison to monthly number of days with laxative administration in October. In contrast, the defecation and laxative administration frequencies did not change after waxy barley consumption among the 14 residents categorized as non-constipated subjects.

**Conclusions:**

Consumption of waxy barley, Kirarimochi, for 5 months improved the bowel movements of the constipated subjects; however, the consumption had no effect on the bowel movements of the non-constipated subjects at Roken nursing home. These results indicate that consuming waxy barley, Kirarimochi, is beneficial for the management of constipation in the late-stage elderly residents at Roken nursing home.

## Background

Development of medical care and socio-economical support for the elderly has resulted in an increase in the human lifespan of many developed countries [1]. The longer late-elderly-stage of individuals has further resulted in concerns: how to maintain a good quality of life (QOL) in this stage defying the senescent dysfunction and physiological alteration. According to the wear-and-tear theory of aging, aging must be accompanied by physiological decline to a greater or lesser degree in late-elderly-stage of individuals [2]. In the Japanese society, 25.9 and 12.5% of the total population were over 65 and 75 years old, respectively, in 2014 [3]. Among the elderly, approximately 1,425,000 people resided in aged care facilities in 2011 [4]. One of the common complaints that exacerbate the residents’ QOL in the facilities is difficult and stressful defecation. This includes straining, hard stools, and feeling of incomplete evacuation, which are mainly because of a decline in the peristaltic movement of the digestive tract. This troublesome situation is usually referred to as “constipation” and is enhanced by common lifestyle factors among the elderly such as a decrease in food and water intake and daily activity [5]. Consequently, late-stage elderly residents at Roken nursing home (a geriatric health services facility [6]) tend to suffer severe constipation that can result in general malaise and clinical manifestations such as colon diverticula [7]. Therefore, control of bowel movements places an additional burden on nursing assistants, and doctors in the facilities tend to prescribe laxatives and/or enema to the affected elderly residents. Such cases are observed on a routine basis in most facilities. For instance, Suyama et al. reported that 79.1% of elderly residents were prescribed some type of laxative to empty the bowels, and 53.2% of the residents were prescribed laxatives of the intestine stimulant type [8]. Such habitual laxative administration may have harmful effects on the intestinal nerve cells and result in a vicious spiral of intractable constipation [9]. The prevention and management of constipation in late-stage elderly residents at nursing homes must be considered from the perspectives of gastrointestinal physiology, geriatric, nursing and care services, and physiological anthropology.

Except for laxative administration, the prevention and management of constipation in elderly residents includes more exercise and consumption of a suitable diet [5]. It is very difficult for the residents at Roken nursing home to manage their bowel movement by exercising more because they are too aged and frail to perform enough exercise that affects bowel movements. The Ministry of Health, Labor and Welfare reports that the average age of nursing home residents is 82.6 years, and 88% of the residents are categorized as being over level three of with respect to the nursing care level [4], and generally, they are disable to walk or to stand up by themselves. Therefore, consuming a suitable diet comprising fiber is the most promising method for the management of residents’ constipation. In the course of our trials for the treatment of constipation in the late-stage elderly residents of a nursing home, we published two papers in the Japanese Journal of Physiological Anthropology: “Effect of ingestion of indigestible dextrin on defecation in late elderly residents in nursing home” [10] and “Effect of lactosucrose ingestion on defecation of late elderly residents in nursing home” [11]. Indigestible dextrin and lactosucrose have been available for healthy adults as effective prebiotics to relieve constipation. However, in our studies, although these saccharides showed a significant positive effect on the improvement of stool characteristics, there was no significant effect on the defecation frequency of the elderly subjects. The most likely reasons for inefficiency of these saccharides on the treatment of constipation were as follows: we administrated these saccharides only for 1 month and only once a day. Thus, a significant effect on the bowel movement of the late-stage elderly residents was not observed.

It is generally accepted that a high-fiber diet increases stool weight and decreases colon transit time [12]; therefore, adequate consumption of a high dietary fiber diet is globally recommended for the prevention and management of constipation [13]. We accordingly tried serving the residents at Roken nursing home with a popular and “healthy” Japanese meal, Mugi-gohan (a boiled mixture of rice and pressed barley), as the main meal several years ago because barley has been shown to be a high-fiber crop [14]. Furthermore, it is a common grain consumed with rice by the Japanese people for many centuries. However, in the trial, the elderly residents tended to lose their appetite for the main meal and would leave it unfinished, resulting in an inadequate calorie intake. The elderly residents stated that the reason for their loss of appetite for consuming Mugi-gohan were that the boiled pressed barley had a disagreeable smell, the texture was not glutinous unlike their favorite rice, and the dark-brown color contrasted to the white color of boiled rice. In another trial, we served the elderly residents with dishes that were prepared using high-fiber foods such as mushrooms, various leafy plants or their leaves, and stems and roots eaten as vegetables (approximately 20 g dietary fiber per day). These dishes also appeared to be unpalatable for the late-stage elderly residents because they could not digest such vegetables easily owing to impaired drinking and eating abilities. Consequently, they tended to leave dishes unfinished, resulting in an inadequate calorie intake.

After the unsuccessful trials, we were informed that a new variety of waxy barley was available for trial if it could relieve constipation in the late-stage elderly residents at nursing homes. This newly bred waxy barley (variety name, Kirarimochi) contains relatively more insoluble and soluble dietary fiber (β-glucan) than other barley varieties and has unique genetic characters such as being proanthocyanidin (*ant28*) and amlylose (*wax*) free [15]. The former genetic character gives the barley a low content of polyphenol so that there is less brown discoloration after boiling with rice. The latter character gives the barley a texture similar to glutinous rice so that the elderly are more likely to accept the texture of Mugi-gohan prepared by boiling rice with this waxy barley (Kirarimochi Mugi-Gohan; KMG). These characteristics would mitigate the discontent with respect to the common pressed barley consumption in the elderly. The advantage of serving KMG in the trial is that the residents could intake an adequate amount of dietary fiber through their daily main meal and not through special meals such as prebiotic solutions or high fiber dishes. This would not burden the nursing assistants with an additional load of work during the preparation of special meals and would enable conducting a long-term trial to examine the effect of waxy barley, Kirarimochi, consumption on the bowel movements of late-stage elderly residents at Roken nursing home.

In this study, we compared the defecation and laxative administration frequencies of the late-stage elderly residents at Roken nursing home before and after KMG consumption in order to examine the effectiveness of the waxy barley consumption for the prevention and management of constipation in the late-stage elderly residents at Roken nursing home.

## Methods

### Subjects

At the beginning of this study, 34 late-stage elderly residents (7 males and 27 females with a mean age of 88.9 ± 7.9 years) were selected as the study subjects. They were able to ingest Mugi-gohan orally and their defecation habits were recorded by nursing assistants to identify those who required sanitary goods and/or toileting assistance. We excluded the residents who were able to defecate by themselves and/or had chronic diarrhea throughout this study. All subjects’ defecations were under the scrutiny of the nursing home doctor and nursing assistants, wherein the nursing assistants administered laxatives to the subjects after the subject had not defecated for three consecutive days until the subject defecated. At the beginning of and during this trial, the nursing assistants had no information or knowledge about the effect of barley consumption on bowel movements; therefore, they had no expectation of an outcome or effect throughout the trial. We explained the purpose of this study and the procedures involved in this study to all the subjects before they gave their written consent for participation. This informed consent was obtained according to the protocol approved by the Research Ethics Committee of Mimasaka University (approval date, August 20, 2015). This approval is based on the principles of the Declaration of Helsinki of the World Medical Association. During the study period, individual health conditions were under medical observation by a nursing home doctor, and no subject experienced suffering severe diarrhea and/or other illness throughout the study period. This experiment was performed from October 2014 to April 2015.

### Experimental protocol and measurements

#### Trial schedule

Table 1 shows the trial schedule showing each period name, type of main meal served in each period, and actual period (to adjust the number of days in one period to be 31 days). In the month before KMG was served, October, all subjects were served boiled rice (Meshi) or rice-porridge as the main meal. The defecation and laxative administration frequencies recorded in October were referred to as *baseline* frequencies to those recorded during the five KMG consumption periods. In this *trial*, we served the subjects with KMG as the main meal for breakfast, lunch, and dinner during the five periods. We prepared KMG by boiling rice with Kirarimochi of rice-grain shape (KRGS) instead of common rolled Kirarimochi, to improve subjects’ consumption of KMG. KRGS is a processed grain produced by cutting and pearling of barley grain, and it has a rice-grain shape so that elderly subjects seldom recognize the presence of barley in KMG. Considering that the risk of diarrhea among subjects is higher because of the adverse effects of consuming KMG, in which the dietary fiber content is much higher than that in boiled rice (Meshi), we served subjects with KMG prepared by boiling rice and KRGS at three different weight ratios; 9:1 (10% KMG) for the first 2 weeks, 8:2 (20% KMG) for the following 2 weeks, and 7:3 (30% KMG) for the following.

**Table 1:** Trial schedule

	Baseline	KMG consumption period					
Name of period	October	November		December	January	February	March
Main meal	Meshi (boiled rice)	KMG (rice KRGS = 9; 1)	KMG (rice KRGS = 8; 2)	KMG^a^ (rice KRGS^b^ = 7; 3)			
Actual period	October 1–31	November 1–14	November 15–December 1	December 2–January 1	January 2–February 1	February 2–March 4	March 5–April 4
Number of days	31	31		31	31	31	31

^a^ *KMG* Kirarimochi-Mugi-Gohan, that was prepared by boiling rice with waxy barley, Kirarimochi

^b^ *KRGS* Kirarimochi of rice-grain shape

#### Food and nutrient intake

Table 2 summarizes the nutrient values of KRGS and three KMGs of different barley contents in comparison with those of rice grain and boiled rice (Meshi). As described in the “Results” section, we served KMG porridge, which had the same nutritional content as that of KMG, to subjects who used to consume rice porridge because of their low eating ability. In addition, we served a specially processed (softened) side dish, the nutritional content of which was the same as that of an ordinary side dish, to the subjects whose eating ability was too low to consume an ordinary side dish. The menu of side dishes served in the *trial* was made according to the diet serving plans without any particular changes in this trial. Average daily nutrition intake per one subject during each period is summarized in Table 3. The record of the subjects’ leftover of the dishes showed that there were no definite differences in the average rate of leftover among the subjects; therefore, there were no definite differences in the average daily nutrition intake per one subject in the trial.

**Table 2:** Nutrition contents of KRGS and three KMGs comparing with those of rice and boiled rice (Meshi)

	KRGS^a^	KMG with a 10% KRGS	KMG with a 20% KRGS	KMG with a 30% KRGS	Rice^b^	Boiled rice (Meshi)^b^
	Per 100 g edible portion	Per one serving^d^	Per one serving^d^	Per one serving^d^	Per 100 g edible portion	Per one serving^d^
Energy (Kcal)	344.0	177.4	176.8	176.2	356.0	178.0
Protein (gram)	7.4	3.1	3.1	3.2	6.1	3.1
Lipid (gram)	1.8	0.5	0.5	0.6	0.9	0.5
Carbohydrate (gram)	78.6	38.6	38.7	38.8	77.1	38.6
Total fiber (gram)	10.2	0.8	1.3	1.7	0.5	0.3
Water-soluble fiber	7.3	0.4	0.8	1.1	0.0	0.0
Water-insoluble fiber	2.9	0.4	0.5	0.6	0.5	0.3
β-glucan^c^ (gram)	6.7	0.3	0.7	1.0	0.0	0.0

^a^Analyzed by Japan Food Research Laboratories except β-glucan content

^b^Standard Tables of Food Composition in Japan 2015

^c^β-glucan content of KMRG was analyzed by Dr. Y. Ichinose (NARO Institute of Crop Science)

^d^One serving was 50 g

**Table 3:** Average daily nutrition intake per one subject during each period

Period^a^	October	November 1–14	November 15–29	November 30–April 4
Main meal	(Boiled rice)	(KMG with 10% KRGS)	(KMG with 20% KRGS)	(KMG with 30% KRGS)
Energy (Kcal)	1507.0	1447.0	1407.7	1471.8
Protein (gram)	60.3	56.6	59.0	63.4
Lipid (gram)	39.0	41.1	33.3	36.6
Carbohydrate (gram)	220.7	207.1	210.7	215.4
Total fiber (gram)	14.6	15.1	16.2	18.6
β-glucan (gram)	0.0	1.0	2.0	3.0

^a^See Table 1

#### Recorded items

Nursing assistants recorded each subjects’ defecation and laxative administration daily were written on recording sheets that have been used in the nursing home for several years. They recorded the following: the approximate time of defecation together with stool size (not weighed but categorized as small, medium, and large size corresponding to the size of one egg, two eggs, and more eggs, respectively). In addition, the approximate time of laxative administration together with the type of laxative and dosage were recorded.

#### Data analyses

All data were expressed as means ± standard deviation (SD). We counted each subjects’ defecation frequency (frequency per day and days with defecation in a period) and laxative administration frequency (number of days with laxative administration; nursing assistants administered laxative once a day) in a period. In this study, we compared the mean number of defecation frequency per day and mean number of days with defecation in a period as well as mean number of days with laxative administration in a period to the baseline observations in October. This statistical comparison was performed using the paired *t* test (two-sided) separately for “group A” and “group B” (categorization of group A and group B, see Table 5). In addition, we compared the quotient of number of days with defecation and number of days with laxative administration in a period by the paired *t* test and by the independent *t* test (two-sided) for group A and group B, respectively. Statistical analyses of categorical items such as ratio of female to male were conducted by the Fisher’s exact test (one-sided). A *p* value of <0.05 was considered to be statistically significant. These analyses were performed using Microsoft Excel add-in software, Ekuseru-Toukei 2015, Social Survey Research Information Co., Ltd., Tokyo, Japan.

## Results

Twenty-eight of 34 subjects completed this study from October to March (two subjects left the nursing home in the middle of March). Table 4 shows the defecation and laxative administration frequencies of each subject in the six periods. According to the defecation frequency per week in October, which was calculated from the defecation frequency in October recorded by the nursing assistants, we classified the subjects into two groups. One group (group A) included the subjects whose defecation frequency was less than three times per week, and the other group (group B) included the subjects whose defecation frequency was more than three times per week in October. This threshold frequency for the classification (three times per week) is consistent with the general definition of constipation as a defecation frequency of less than three times per week [5]. Table 5 summarizes the statistical comparison of eight items between the two groups: mean age, ratio of female to male, mean nursing care level, ratio of type of supplied main meal, ratio of type of supplied side dish, mean number of days with defecation, mean number of days with laxative administration, and mean quotient of defecation/laxative administration days. Statistical comparison indicates that there were no significant differences in the mean age and the gender composition, whereas the nursing care level was significantly different between the two groups. Therefore, we separately conducted statistical analyses on the defecation and laxative administration frequencies in group A and group B. Table 6 shows a statistical comparison of four items among the subjects in group A. This table clearly shows that the mean number of defecation frequency as well as days with defecation per a period increased in the five periods (the number of days with defecation significantly increased in November, January, and March) in comparison with those observed in October. In addition, Table 6 shows the mean number of days with laxative administration decreased in the five periods (significantly decreased in December and February) in comparison with those observed in October. Consequently, the mean quotient of the days with defecation and laxative administration increased in the five periods (significantly increased in December, January, and February) in comparison with those observed in October. These facts described above indicate that the bowel movements of the subjects in group A improved after consuming KMG as their main meal. In other words, waxy barley consumption had positive effects on bowel movements of the constipated residents at Roken nursing home. In contrast, Table 7 shows that KMG consumption did not affect the defecation and laxative administration frequencies of the residents in group B, i.e*.*, those who needed more nursing care and were regarded as “not constipated.”

**Table 4:** Defecation and laxative administration frequencies of each subject during the trial

ID^a^	Defecation (frequency)							Days with defecation						Days with laxative administration					
	Baseline	Barley consumption period					Per week^b^	Baseline	Barley consumption periods					Baseline	Barley consumption periods				
	October	November	December	January	February	March	October	October	November	December	January	February	March	October	November	December	January	February	March
1	7	13	11	20	18	17	1.6	7	10	9	15	15	14	5	7	3	3	2	3
2	7	11	8	12	6	11	1.6	6	11	7	10	5	7	6	2	4	2	4	4
3	11	13	7	10	9	7	2.5	7	7	6	8	8	4	6	7	5	4	8	12
4	12	11	19	18	19	18	2.7	10	9	14	13	14	13	5	4	2	3	1	5
5	8	5	5	6	15	19	1.8	6	5	5	5	7	17	11	8	9	11	4	8
6	11	7	11	6	17	8	2.5	7	7	7	5	12	9	6	6	6	7	1	4
7	10	12	12	17	11	16	2.3	9	9	9	10	12	13	7	4	3	6	1	4
8	5	7	3	6	4	4	1.1	4	6	3	5	3	4	8	7	4	10	8	9
9	7	7	12	15	13	10	1.6	7	7	5	10	6	7	9	5	6	4	7	6
10	11	13	11	15	11	16	2.5	9	12	9	15	12	14	3	4	0	2	0	1
^c^11	9	31	16	8	16	-	2.0	8	15	12	8	12	-	14	3	3	4	3	-
12	9	11	8	13	7	11	2.0	7	10	8	12	6	11	3	3	4	2	6	1
13	12	15	10	11	9	11	2.7	7	8	8	6	5	6	5	7	4	7	6	6
14	10	9	9	8	5	9	2.3	6	9	6	6	6	6	8	9	5	9	7	8
^d^15	16	17	11	20	13	-	3.6	11	16	14	8	10	-	2	4	2	4	2	-
16	14	11	5	14	12	18	3.2	15	13	15	17	13	15	0	0	3	0	3	2
17	31	37	32	29	16	18	7.0	12	14	10	9	12	12	2	2	2	2	2	2
18	23	24	23	33	23	23	5.2	13	8	11	10	11	10	3	7	4	4	5	4
19	38	42	30	36	27	26	8.6	13	12	8	13	11	9	4	1	4	3	2	6
20	24	20	38	35	26	28	5.4	13	8	5	14	10	15	1	4	3	1	1	4
21	14	11	6	10	1	11	3.2	14	22	15	16	13	12	4	0	2	3	4	7
22	27	21	27	28	35	32	6.1	20	18	18	19	18	19	1	1	1	0	0	0
23	19	15	15	20	14	23	4.3	20	25	20	21	18	17	4	0	0	1	4	3
24	14	16	17	16	23	16	3.2	17	14	21	20	18	17	4	7	3	3	2	3
25	19	19	21	8	14	17	4.3	13	10	6	8	1	10	3	3	3	6	4	0
26	18	17	18	23	22	17	4.1	18	16	19	17	23	21	0	0	0	2	0	0
27	14	17	12	9	17	15	3.2	15	10	10	18	12	18	1	4	3	1	2	0
28	16	11	12	16	14	15	3.6	13	12	15	16	16	14	2	3	1	0	1	3

^a^Subjects from ID 1 to ID 14 belonged to group A where subjects’ defecation frequency was less than 3 times a week in October. Subjects from 15 to 28 belonged to group B where subjects’ defecation frequency was more than 3 times a week in October

^b^Calculated by frequency in October × 7/31 days

^c^The subject of ID 11 left the nursing home on March 10th (no data)

^d^The subject of ID 15 left the nursing home on March 24th (no data)

**Table 5:** Comparison of the subjects’ characteristics between group A and group B

Group	Group A	Group B	*p* value
Weekly defecation frequency in October	Less than 3 times	More than 3 times	
Mean age (the youngest–the oldest)	90.4 ± 8.4 years (65–100 years)	87.4 ± 1.8 years (87–96 years)	0.3071^b^
Numbers of female and male	11 Females and 3 males	10 Females and 4 males	0.5000^a^
Mean nursing care level	2.6 ± 1.2	3.5 ± 1.0	0.0468^b^
Types of supplied main meal	10 Boiled rices and 4 rice porridges	7 Boiled rices and 7 rice porridges	0.2200^a^
Types of supplied side dish	9 Ordinary side dishes and 5 soften side dishes	4 Ordinary side dishes and 10 soften side dishes	0.0642^a^
Mean days with defecation (in October)	7.1 ± 1.5	14.8 ± 2.9	<0.0000^b^
Mean days with laxative administration (in October)	6.9 ± 3.0	2.2 ± 1.5	<0.0000^b^
Mean quotient of defecation days/laxative administration days	1.3 ± 0.7	7.6 ± 5.4	0.0002^b^

^a^Compared by the Fisher’s exact test (one-sided)

^b^Compared by the independent *t* test (two-sided)

**Table 6:** Statistical comparison of defecation and laxative administration frequencies, and the quotient in group A

	Period^a^	October	November	December	January	February	March
	*n*	14	14	14	14	14	13^b^
Defecation frequency/period	Mean	9.2 ± 2.2	11.8 ± 6.3	10.1 ± 4.1	11.8 ± 4.7	11.4 ± 5.0	12.1 ± 4.7
	*p* value (two-sided)^c^		0.145	0.35	0.074	0.117	0.051
Days with defecation/period	Mean	7.1 ± 1.6	8.9 ± 2.6	7.7 ± 2.9	9.1 ± 3.6	8.8 ± 3.9	9. 6 ± 4.3
	*p* value (two-sided)^c^		0.014	0.252	0.024	0.056	0.030
Days with laxative administration/period	Mean	6.3 ± 2.3	5.4 ± 2.1	4.1 ± 2.1	5.5 ± 3.2	4.1 ± 2.9	5.5 ± 3.2
	*p* value (two-sided)^c^		0.146	0.003	0.087	0.018	0.248
Days with defecation/days with laxative administration	*n*	14	14	13^d^	14	13^d^	13^b^
	Mean	1.3 ± 0.7	2.1 ± 1.5	2.2 ± 1.8	2.8 ± 2.3	4.4 ± 5.1	3.5 ± 4.2
	*p* value (two-sided)^c^		0.061	0.033	0.007	0.033	0.052

^a^Period; see Table 1

^b^One subject left the nursing home in March

^c^Compared to that in October by the paired *t* test (two-sided)

^d^Excluded one subject with no laxative administration

**Table 7:** Statistical comparison of defecation and laxative administration frequencies, and the quotient in group B

	Period^a^	October	November	December	January	February	March
	*n*	14	14	14	14	14	13^b^
Defecation frequency/period	Mean	20.5 ± 7.3	19.9 ± 9.2	19.1 ± 9.9	21.2 ± 10.0	18.4 ± 8.3	19.5 ± 6.4
	*p* value (two-sided)^c^		0.521	0.381	0.650	0.292	0.515
Days with defecation/period	Mean	14.8 ± 2.9	14. 1 ± 5.0	13.4 ± 5.2	14.7 ± 4.5	13.3 ± 5.2	14.5 ± 3.8
	*p* value (two-sided*)* ^c^		0.568	0.172	0.921	0.163	0.428
Days with laxative administration/period	Mean	2. 2 ± 1.5	2.6 ± 2.4	2.2 ± 1.3	2.1 ± 1.8	2.3 ± 1.5	2.6 ± 2.3
	*p* value (two-sided)^c^		0.615	1.000	0.873	0.850	0.457
Days with defecation/days with laxative administration	*n* ^d^	12	10	12	11	10	9
	Mean	7.6 ± 5.4	5.6 ± 5.4	6.4 ± 5.2	8. 0 ± 6.7	5.2 ± 2.9	4.0 ± 2.1
	*p* value (two-sided)^e^		0.407	0.585	0.858	0.226	0.107

^a^Period; see Table 1

^b^One subject left the home in March

^c^Compared to that in October by the paired *t* test (two-sided)

^d^Excluded subjects with no laxative administration

^e^Compared to that in October by the indenpendent *t* test (two-sided)

## Discussion

It should be noted that this trial was conducted at Roken nursing home; its mission is “to enable a person under a condition of need for long-term care to live a long and meaningful life” [9], and its residents are the late-stage elderly whose ADL (activities of daily living) has weakened. Most subjects in this study were over 80 years old, and their nursing care level (which reflects ADL weakness) was from level 2 to level 5 (level 2; those who need assistance to have a meal and defecation, level 5; those who cannot have a meal and defecation by themselves). As described in the “Results” section, we classified the subjects into two groups, group A and group B, according to the weekly defecation frequency in October. As shown in Table 5, the mean nursing care level of the subjects in group B was significantly higher than that of the subjects in group A, i.e., the ADL of the subjects in group B was relatively lower compared with that of the subjects in group A. Considering the relationship between nursing care level and eating ability of the elderly, it is generally accepted that the elderly with a higher care level tend to have poorer eating ability [16]. As described in the “Food and nutrition” section, subjects whose eating ability was low consumed rice porridge with ordinary dishes or softer foods depending on their degree of eating ability, poorer eating ability of the subjects in group B compared with group A was indicated by the larger number of subjects who consumed softened dishes in group B than in group A; ten out of fourteen subjects in group B consumed softened dishes, whereas only five in group A consumed the same (Table 5). The level of ADL and eating ability may influence the function of the intestinal tract; therefore, it made sense to conduct statistical analyses of the defecation frequency together with the laxative administration frequency of the subjects in group A separately from those in group B to truly ascertain the effect of consumption of waxy barley, Kirarimochi, on the bowel movements of the late-stage elderly residents at Roken nursing home.

First, we discuss the effect of waxy barley, Kirarimochi, consumption on the bowel movements of subjects belonging to group A. Table 6 clearly shows that consumption of KMG improved bowel movements of the subjects in group A; the mean defecation frequency and the mean number of days with defecation in a period increased; consequently, mean number of days with laxative administration in a period decreased under nursing assistants’ control. For example, mean monthly defecation frequency in March was 12.1 ± 4.7 times (i.e., 2.7 ± 1.1 per week), whereas that in October was 9.2 ± 2.2 times (i.e., 2.1 ± 1.1 per week). This increased mean defecation frequency in March (2.7 times per week) was close to the threshold defecation frequency of the general definition of constipation (three times per week) indicating that the subjects could maintain their bowel movement at nearly normal levels having less frequent laxative administration. In addition, the quotient of defecation and laxative administration days indicated the effectiveness of one laxative administration on bowel movements. For instance, the quotient was a mean of 1.1 in October, which means the subjects had 1 day with defecation after one laxative administration. The quotient significantly increased to 4.4 on average in February, which means the subjects had 4 or 5 days with defecation after one laxative administration. Considering that most subjects were of atonic constipation and prescribed with a laxative that irritated the intestinal tract, consumption of waxy barley, Kirarimochi, could improve intestinal activity such as colonic peristaltic motion. The exact mechanism of this waxy barley on the improvement of the colonic peristaltic motion has been reported by De Angelis et al. [17]. They studied the effect of whole-grain barley on the human fecal microbiota and metabolome and found that barley appeared to be effective in modulating the composition and metabolic pathways of the intestinal microbiota, leading to an increased level of short-chain fatty acids (SCFA) that stimulate the colonic peristaltic motion.

It is generally accepted that adequate consumption of a high dietary fiber diet is beneficial for relief of constipation [5, 12]. However, Kenny and Skelly conducted a systematic review on eight studies and showed that the effectiveness of dietary fiber for the management of constipation has not been established in older adults who reside in nursing homes or long-stay homes [18]. Their systematic review describes treatments with dietary fibers such as corn, wheat bran, and high-fiber cookies at fiber dose (g/day) from 3.3 to 10.0, whereas treatment duration varied from 2 to 13 weeks. In our present study, we found that long-term consumption (5 months) of a boiled mixture of rice and waxy barley, Kirarimochi, improved the bowel movements of constipated elderly subjects at Roken nursing home. This suggests that waxy barley consumption is beneficial for the management of constipation in older adults. The difference between the studies described by Kenny and Skelly and our present study include the amount of daily intake of dietary fiber and treatment duration. The daily intake of dietary fiber in the present trial was 15.1, 16.2, and 18.6 g of total dietary fiber when served with KMG with a 10% KRGS, KMG with a 20% KRGS, and KMG with a 30% KRGS, respectively (Table 3). The values are much higher than those reported by Kenny and Skelly (from 3.3 to 10.0 g per day). In addition to high dietary fiber content of KMG, KMG with a 30% KRGS serving could provide the subject with soluble barley dietary fiber (β-glucan) at the dose of 3 g per day (Table 3). Soluble dietary fiber, β-glucan, has been proven to be a functional barley polysaccharide that has many beneficial effects on human health [19], including increased production of SCFA in the colon. It is noteworthy that daily consumption of β-glucan in the present study (3 g per day) meets the minimum daily intake of barley β-glucan recommended by Food and Drug Administration of the USA to reduce coronary heart disease risk [20]. Regarding the long treatment duration of our present study, we believe the following two points highlight how the study was completed over 6 months. We avoided burdening the nursing assistants with additional duties in preparation of the meals by serving the subjects with their daily main meals that were high in dietary fiber. Consequently, we could obtain defecation and laxative administration data, which fulfilled the criteria for statistical analyses through written recording sheets. Another point is that the elderly subjects were receptive to Mugi-gohan prepared from rice and waxy barley, Kirarimoch, of rice-grain shape. Their receptiveness to Mugi-gohan was confirmed by the fact that no subject left meals unfinished during the experiment period, and this palatability may help to prevent subjects from dropping out of this long term study.

Second, we consider the effect of waxy barley, Kirarimochi, consumption on the bowel movement of the subjects belonging to group B. In contrast to the positive effect of the barley consumption on the bowel movements of the constipated subjects in group A described above, barley consumption had no effect on the bowel movements of subjects in group B. Table 7 shows that the mean number of days with defecation in any period did not vary significantly even after the subjects were served KMG. Consequently, the mean number of days with laxative administration in any period did not significantly vary. These facts indicate that waxy barley, Kirarimochi, consumption caused neither too frequent defecation nor diarrhea, which we were feared before the dietary intervention. We speculate that the composition of dietary fiber of the waxy barley, Kirarimochi, which has higher soluble fiber than insoluble fiber (Table 2), resulted in neither a negative nor positive effect on bowel movements of the subjects whose ADL and eating ability were poor. This “neutral” effect of the consumption is helpful for food service management in nursing homes because the dining room does not need to prepare two kinds of meals containing barley, one for constipated residents and another for non-constipated residents.

Last, we should mention the limitation of the present study, which was that it was not a double-blinded, randomized control trial. The main reason why we designed this trial as an observational study not a case-control study (in which subjects in a control group consumed boiled rice) was the following. The Roken nursing home (a geriatric health services facility) where we conducted this trial is a public health services facility for local citizens and its mission statement is “to enable the *all* facility residents under a condition of need for long-term care to live a long and meaningful life”. Therefore, from public and ethical point of view, we could not “select” some facility residents as the subjects who consumed boiled rice instead of waxy barley that had been proved to have some health benefit to the consumer. We had to treat all the facility residents equally; in this case, we had to serve all the residents with the same meals that contained waxy barley. One more reason is the following: The working environment of public nursing home is getting hard in recent years, and the numbers of nursing assistants and dining room staffs are limited. It would be an additional burden for the nursing assistant to serve the residents of two different groups, such as a control group and a case group, with two different meals. And it would be also an additional burden for dining room staffs to prepare two different meals, boiled rice and KMG, as the residents’ everyday meals. They are too busy to do additional work that would be predicted by applying a double-blinded, randomized control study design.

## Conclusions

Five-month trial, where the subjects consumed meals of mixed boiled rice and waxy barley, Kirarimochi, instead of boiled rice only, improved the bowel movements in the constipated residents, whereas no effect was observed on the bowel movements of the subjects who needed more nursing care and were regarded as “not constipated” at Roken nursing home. These results indicate that the consumption of waxy barley, Kirarimochi, is beneficial for the management of constipation of the older adults living in Roken nursing home.

## Abbreviations

ADL: Activities of daily living
KMG: Mugi-Gohan that was prepared by boiling rice and waxy barley
KRGS: Kirarimochi of rice-grain shape
QOL: Quality of life
